# Regulating for uncertainty: bridging blurred boundaries in medical innovation, research and treatment

**DOI:** 10.1080/17579961.2019.1573400

**Published:** 2019-02-03

**Authors:** Nayha Sethi

**Affiliations:** a Mason Institute, University of Edinburgh, Edinburgh, UK; b Centre for Biomedicine, Self and Society, Usher Institute, University of Edinburgh, Edinburgh, UK

**Keywords:** Research, treatment, innovation, health research, regulation

## Abstract

This paper explores the blurred conceptual boundaries between ‘practice/treatment’, ‘research’ and ‘medical innovation’ in order to inform what these mean – and can mean – for regulation of these fields of enquiry. These terms are constantly employed within the sphere of health and human health research regulation, but there is a lack of clarity and consistency in the ways in which the activities are categorised. This gives rise to confusion and can negatively impact treatment/research and innovation. I argue that it is not only timely but also necessary to revisit our current conceptualisations of these key activities, with a particular emphasis on medical innovation. The proposal is to reimagine regulatory landscapes – including regulation – in more holistic terms that reflect the processes that transgress these categories and to understand better the blurred boundaries that exist between them. I suggest that the anthropological concept of liminality is particularly helpful in developing more holistic understandings of medical innovation that reflect the processes and relationships that exist. Importantly, it provides us with a new conceptualisation of medical innovation as a shared space where both practice/treatment and research can co-exist.

## 1. Introduction

In 1979, pioneering health law academic Bernard Dickens noted:

A medical profession which did not seek improved means to conquer disease would be condemned for dereliction of its duty. Members of the public will not accept the current state of the medical arts as finite but feel justified in expecting the development of more effective therapies for illness, and the promotion of improved means of preventive care.^1^

Four decades later, for some 10,000 known health problems, we currently only possess around 500 viable treatments.^2^ It could be argued that the pressures to provide treatment, conduct research and innovate not only remain, but are stronger than ever. Technological developments have increased our understanding of disease, and there has been a marked growth in public awareness of, and engagement with, health related issues.^3^ For example, social media is emerging as an avenue through which patients may even seek to participate in research and access experimental therapies, often under expanded access^4^/ compassionate use programmes.^5^ This comes hand in hand with a swathe of ‘Right to Try’ legislation adopted across many United States jurisdictions.^6^ In the UK, proponents of the Medical Innovation Bill (the Saatchi Bill), which provoked lively debate,^7^ argued that innovation was stifled due to the current regulatory landscape.

In the public health sphere, the West-African Ebola and Zika viruses have highlighted the ongoing need to rapidly develop novel therapies where effective cures do not already exist. Numerous initiatives are under way in order to decrease the time lag between identification of public health emergencies and availability of effective treatments via research and innovation.^8^ For example, the UK government has partnered with Innovate UK in investing £35 million towards the development of new vaccines for diseases of epidemic potential^9^ and policy pushes towards innovation appear in other translational fields such as regenerative medicine.^10^ The recently formed Accelerated Access Collaborative^11^ was introduced in order to streamline and speed up patient access to innovations. Considerable economic commitments towards boosting innovation are also afoot at the European level^12^ and the Commission's strategy for Responsible Research and Innovation is noteworthy.^13^

However, alongside the drive for health improvement through innovation come significant challenges calling into question the efficacy of pre-existing regulatory approaches. This article considers some ways in which current conceptualisations of medical innovation are problematic. I suggest this is so not only due to ambiguities surrounding what might constitute medical innovation, but also due to the blurred, overlapping boundaries existing between medical innovation, practice/treatment, and research. It is my claim that existing conceptualisations fail to adequately account for many of the nuances, diverse processes and experiences associated with the activities. A more holistic account of medical innovation – and related activities – is much needed. The building blocks of such an account are a processual approach to regulation, examining regulatory spaces and experiences of actors and relying upon the anthropological concept of liminality.^14^

Examples included in the discussion will highlight the pressing need to achieve conceptual clarity in our regulatory characterisations. Paradoxically, this clarity might be achieved not necessarily through further categorisation of activities – which can represent a typical regulatory response towards areas of uncertainty,^15^ but rather, through collapsing distinctions and exploring grey areas of overlap. One example is the learning healthcare system, an environment in which ‘knowledge generation is so embedded into the core of the practice of medicine that it is a natural outgrowth and product of the healthcare delivery process and leads to continual improvement in care’.^16^ The increasing prevalence of such systems demonstrates growing difficulty in distinguishing between processes respectively categorised as ‘practice/treatment’ on one hand and ‘research’ on the other.^17^ Identifying the most appropriate level of ethical oversight becomes challenging and confusion can be exacerbated by the duality of (potentially conflicting) roles played by different actors in the healthcare setting such as the ‘clinician-investigator’ and the ‘patient-participant’. The Access to Medical Treatments (Innovation) Act 2016 and, in particular, proposals to establish a register for innovative treatments are also considered. The register provides a clear example of how a liminal approach might help us to better navigate areas where roles and activities are overlapping. The recent scandal involving thoracic surgeon Paolo Macchiarini and his ‘fatal experiments’^18^ is also discussed. One of the key findings from an inquiry into the operations, discussed below, was that there was difficulty in determining whether Macchiarini and his teams’ activities represented clinical care or research.^19^ Further, ‘medical innovation’, despite its pervasiveness within the health setting, remains ill-defined; its relationship to, and differentiation from, practice/treatment and research remains obscure, and in need of further unpacking. The intention here is not to provide a thorough analysis of the various and diverse definitions of medical innovation (and relatedly, practice/treatment and research). Nor is it to advance specific suggestions as to regulatory mechanisms which ought to be employed for the regulation of medical innovation. Rather, through brief consideration of these activities and the use of several examples, the discussion sheds light on why we need to, and how we might, develop a richer, more holistic conceptualisation of medical innovation and related activities. This is important because before we can determine whether we are using the most appropriate regulatory approaches to govern activities, we first need to ensure that we understand the nature of the activities which we seek to regulate.

The remainder of this article is structured as follows. I provide an overview of typical conceptualisations of practice/treatment and research (Part 2) and medical innovation (Part 3) and the regulatory frameworks that each engage; such an overview demonstrates that the boundaries between these activities are blurred, and that regulation needs to better acknowledge this. Part 4 argues that the anthropological concept of liminality is of value in developing a more holistic approach towards conceptualising these activities. Such conceptualisation better accounts for the grey areas of overlap between practice/treatment and research and the processes and experiences associated with them, and thus might contribute towards improved regulation of these activities.

## 2. Current conceptualisations and blurring boundaries between practice/treatment and research

Although the primary focus of this article is on medical innovation, it is necessary initially to consider conceptualisations of practice/treatment and research for two reasons. First, a significant underappreciated point is that medical innovation is inextricably linked to those activities typically characterised as practice/treatment and research, to the extent that it may not be clear where one activity ends and the other begins. Thus, in order to better understand medical innovation and the associated regulatory challenges, we must also consider practice/treatment and research. Secondly, the long-noted problematics of the distinction between practice/treatment and research can helpfully be contrasted with the further complexities which medical innovation brings to the regulatory sphere. For the purposes of this article, the terms ‘practice’ and ‘treatment’ are used synonymously and referred to as ‘practice/treatment’. Whilst it is acknowledged that nuances between ‘practice’ and ‘treatment’ might exist,^20^ for present purposes it is sufficient to contrast practice/treatment together, with research and innovation.

It is well established that the traditional locus of the distinction between practice/treatment and research centres on the intention and design of an activity. Whilst the Belmont Report is better known in the US as a foundational document which advances ethical principles for biomedical research, it also provides definitions of medical practice as:^21^

interventions that are designed solely to enhance the wellbeing of an individual patient or client and that have a reasonable expectation of success. The purpose of medical or behavioral practice is to provide diagnosis, preventive treatment or therapy to particular individuals.^22^

In contrast, ‘research’:

designates an activity designed to test an hypothesis, permit conclusions to be drawn, and thereby to develop or contribute to generalizable knowledge (expressed, for example, in theories, principles, and statements of relationships). Research is usually described in a formal protocol that sets forth an objective and a set of procedures designed to reach that objective.^23^

Thus, the primary intent in practice/treatment is to provide benefit to the individual patient, whereas research aims to contribute to the stock of generalisable knowledge, normally through adherence to a research protocol. But even the authors of the Belmont Report acknowledged explicitly *within* the Report, drafted forty years ago, the difficulty in differentiating between these activities, ‘partly because both often occur together’.^24^ Despite this recognition of overlap, these definitions and the basis for distinction between practice/treatment and research have been widely replicated, and continue to persist across health research regulation.^25^ Let us briefly consider how practice/treatment and research are broadly regulated in the UK.^26^

A substantial body of case law exists around the standard of care expected from doctors within the traditional doctor-patient relationship. In short, such determinations generally turn on failure on the part of a doctor to act ‘in accordance with a practice accepted as proper by a responsible body of medical men skilled in that particular art’.^27^ This is one of the elements that could lead to a civil claim against a doctor in medical negligence, or indeed, in the most serious cases, to a criminal charge of gross negligence manslaughter where a patient subsequently dies. Various considerations will impact on standard treatment, including current guidance from the Royal Colleges.^28^ The National Institute for Health Care and Clinical Excellence (NICE) is charged with providing recommendations and guidance on which treatments ought to be provided to patients through the NHS.^29^ Doctors can consult this guidance and exercise their judgement around whether to prescribe treatments to a patient. All doctors in the UK must be registered with the General Medical Council (GMC), the statutory regulator for doctors in the UK, regardless of the setting in which they work. Doctors must use their professional judgment to adhere to ethical guidance provided by the GMC,, including ‘Good Medical Practice’, which sets out the professional values, knowledge, skills and behaviours expected of all doctors working in the UK.^30^ Where concerns are raised about a doctor's conduct, the GMC has the power to investigate these and may refer cases to its adjudicatory arm – the Medical Practitioners Tribunal Service – which can impose restrictions on a doctor's ability to practise.

In contrast, researchers wishing to conduct studies involving human participants are, by the earlier definition, seeking to advance knowledge, rather than apply standard treatments to patients on an individual basis. Permissions must be sought before this is carried out, such as ethical approval(s).^31^ Depending on the type of research in question, approvals must be sought from a Research Ethics Committee. Additional approvals may be required from the relevant regulatory authority. For example, for health and social care studies involving the NHS (and being led in England), approval must be sought from the Health Research Authority.^32^ The Medicines and Healthcare products Regulatory Agency is responsible for regulating medicines and medical devices in the UK, including approving clinical trials, and providing market authorisation and licences for pharmaceuticals.^33^ In turn, each type of research engages various European regulations.^34^ Whilst regulation of research will depend upon the type of research engaged, there is no overarching statutory regulation of research in the UK. As such, the relevant research organisations are normally charged with investigating claims of scientific misconduct, the UK Research Integrity Office plays an advisory role in such investigations.

The regulation of practice/treatment v research may appear sufficiently distinctive so as to imply that discerning between them is relatively straightforward. But numerous areas of regulatory overlap exist. For example, the GMC is responsible for regulating doctors by virtue of their profession, regardless of whether they are carrying out medical practice/treatment or research.^35^ The Human Tissue Authority regulates those organisations which remove, store and use human tissue for research *as well as* for treatment. Further, technological developments in how medicine is practised, treatments offered, and research conducted, mean it may be harder than ever to delineate between the activities and to determine important regulatory considerations. These include: determining which activities should be subject to ethical oversight, which level of oversight and by whom; questions of legal liability; what standard of care actions might be judged against; and what can be done with information gathered in the course of the practice/treatment or research. The ‘learning healthcare system’ draws practice/treatment and research closer together ‘by building knowledge development and application into each stage of the healthcare delivery process’.^36^ Kass and others note that these ‘deliberately integrated’ systems lead to increasingly blurred distinctions between practice/treatment and research. A regulatory consequence is that this leads to ‘overprotection of the rights and interest of patients in some cases and to underprotection in others’.^37^ For example, patients may be exposed to significant risks in clinical practice/treatment via interventions of unproven clinical benefit and risk, performed by emergency surgical teams who have never or only rarely performed a procedure whereas low-minimal risk observational studies (research) must undergo prior ethical review.^38^ Challenges are also prevalent in the public health emergency setting where overlap is especially pronounced between activities focused on treating individuals and those aimed at rapidly conducting research in order to develop novel treatments. An on-going theme is the mismatch between frameworks governing public health research and treatment.^39^ Again, regulatory challenges exist despite the important implications which each classification can have in terms of whether activities necessitate ethics review.^40^ In the emergency setting, the only potential ‘treatment’ option available may be through participation in trials which are ultimately designed to generate generalisable knowledge and where participants may not necessarily be assigned the investigative agent, but may be assigned to a control arm. Indeed, identifying and obtaining appropriate ethics approval for experimental vaccines during the Ebola epidemic proved problematic, leading to substantial delays when timely response was crucial. ^41^

Another example can be found in the context of genomics. Due to the gap in translation between (1) early adoption of technologies in the research setting and (2) their implementation in the clinical care setting, it has been suggested ‘clinical-esque’ obligations have become incumbent upon genomic researchers. Questions arise around potential responsibilities to report incidental findings which are ‘discovered in the course of research that are beyond the aims of the study’.^42^ All of these concerns can impact the standard of care potentially owed to patients/research participants and give rise to uncertainty on the part of clinicians/researchers with regards to the nature and scope of these potential obligations.^43^ Numerous additional examples of the challenges and blurring boundaries between practice/treatment and research are prevalent across a wide range of settings.^44^ And, as considered next, ‘innovation’ adds further complexity.

## 3. Further complexities: medical innovation

Innovation gives rise to varying definitions;^45^ this is surprising given: the frequency with which the term is used; the considerable commercial investments towards boosting innovation;^46^ and the commitment towards understanding how we can foster ‘responsible research and innovation’.^47^ On the other hand, this ambiguity is not surprising given the inherent open texture of language^48^ and the variety of different settings (e.g. health, finance, agriculture) in which innovation occurs.^49^ Butenko and Larouche propose three common elements of innovation: ‘(i) an invention (ii) which is diffused and adopted and (iii) which has a positive social impact’^50^ (albeit that there is also potential for negative impact).^51^ In turn, each of these components is subject to varying interpretation on, for example: what constitutes an invention (including what level of ‘newness’ is required); what constitutes diffusion and adoption and what constitutes a positive social impact. Further, and of particular significance to the discussions which will follow, it has been stressed that (iv) innovation may also be conceptualised as a process^52^ not just a product.

Moving to innovation in the medical context, diverse conceptualisations of medical innovation are present, again, despite the frequency with which the term appears across the health landscape.^53^ The regulatory framework governing ‘innovative treatments’^54^ in the UK is piecemeal.^55^ As with standard medical treatments, NICE is charged with assessing whether innovative treatments ought to be approved for NHS use. NICE issues guidelines which inform decision-making within NHS Clinical Commissioning Groups subject to each group's priorities and discretion on the part of clinicians. But further categories of access to ‘innovative treatments’ also exist. Compassionate Use Programmes (CUPs)^56^ are designed to provide patients with life-threatening illnesses early access to unproven interventions (unauthorised medical products which have not yet received market authorisation).^57^ Such access is typically limited to situations where no current treatment exists and where individuals may not fit the clinical trial participation criteria. A further avenue for accessing untested interventions is via ‘hospital exemptions’ allowing for one-off manufacturing of Advance Therapy Medicinal Products (ATMPs). These comprise of gene therapy medicinal products (GTMPs), somatic cell therapy medicinal products (sCTMPs), tissue-engineered products (TEPs) and combined ATMPs. Clinicians must apply to the MHRA for a manufacturer licence to use the ATMP for a specific patient.^58^

Categorising innovation under each of these activities renders them subject to distinct regulatory oversight with significant variation (and uncertainty) around patients’ access to the innovation, acceptability of levels of risk-benefit analysis, potential liability^59^ and requirements for evidence gathering.^60^ In addition, different countries have different regulatory frameworks for each type of innovation, a point of concern at a time of increased medical tourism^61^ and growing internationalisation of research.^62^ For example, there are internationally varying processes and standards for, amongst others, Research Ethics Committees/Institutional Review Boards to provide prior ethical review for compassionate use,^63^ use of autologous stem cells^64^ and varying interpretations and applications across EU Member States of the term ‘non-routine’ for the purposes of hospital exemptions.^65^ The complexity is exacerbated by additional terms associated with innovation, for example ‘experimentation’, ‘experimental therapy’, ‘innovative treatment’ and ‘innovative therapy’.^66^

Some work has been dedicated to offering context-specific definitions of medical innovation, including in the fields of surgery,^67^ autologous stem cells^68^ and drug therapy.^69^ One common feature is departure from standard treatment but the extent of departure required to warrant ‘innovation’ varies. For example, Section 2(2) of the Access to Medical Treatments (Innovation) Act 2016^70^ (‘AMTIA’) defines innovative medical treatment as ‘medical treatment for a condition that involves a departure from the existing range of accepted medical treatments for the condition’. Such treatments, per Section 3(2)(a) and (2)(b) include ‘amongst other things’ off-label use of authorised medicinal products and medicinal products with no marketing authorisation. Beyond these specifications and acknowledgement that innovation can involve both processes and products, the Act offers little detail. Chan suggests innovative treatment/therapy represents ‘*significant* departures from standard medical therapy which has not been validated by reliable research methods, or where there is simply insufficient evidence to support the safety and efficacy of the innovative procedure, method, or device’.^71^ Another definition suggests that these are ‘uncontrolled, often single, interventions intended to manage or solve particular problems’ which are not carried out in order to add to the stock of knowledge but primarily to benefit the individual.^72^ It could be argued that these descriptions suggest medical innovation is primarily directed at benefitting individual patients and thus neatly falls under the category of practice/treatment, but this may be because the terminology used i.e. innovative *therapy*, innovative *treatment*, innovative *practice* already imply a practice/treatment-centric goal of individual benefit. But a counter implication is that research may be more aligned to innovation on the basis of its contribution to the development of new knowledge. Medical innovation is often the starting point upon a translational continuum which, through on-going research and development,^73^ can lead to the introduction and adoption of an innovation which may eventually become a standard practice/treatment option.^74^ Thus innovation can straddle *both* practice/treatment and research, ‘occupying a position somewhere between “standard practice” and “clinical research”’.^75^ As I suggest further below, innovation can, then, be conceived of as a space within which activities focussed towards both individual *and* societal benefit can co-exist. Indeed, normatively, this is what we would wish to happen. But how can we make better sense of these overlapping spheres of action with interchanging actors? It is here we find value in the anthropological concept of liminality. Liminality is precisely concerned with spaces ‘in between’. In the next section, I therefore explore how the concept can provide a helpful analytical lens through which to explore those spaces occupied by medical innovation and relatedly, practice/treatment and research.

## 4. Liminality, process and experience: achieving conceptual clarity via blurring boundaries?

Further clarity around medical innovation could be achieved by offering more specific and prescriptive descriptions of what each activity constitutes. However, this could lead to added complication as the law's tendency to categorise activities and objects of regulation is invariably challenged by, for example, the rapid pace at which new technologies develop^76^ and new objects of regulation are created. The effect of this tendency to categorise has been characterised as regulatory compression: ‘a silo-based approach to classifying research objects that: (1) limits the flexibility necessary in clinical and laboratory research; and (2) results in the emergence of unregulated spaces that lie between the bounded regulatory spheres’.^77^ Indeed, this categorisation can often take place via the introduction of more rules which may not always represent the most appropriate regulatory response.^78^ By exploring how medical innovation might overlap with other categories of activity, such as the practice/treatment-research divide, I am not suggesting that classification and distinctions are not necessary or important, nor am I dismissing the value of pre-existing definitions which have been offered in various contexts. Rather, an emphasis on overlap reveals areas of *meaningful* distinction and true differentiation between them and reduces the likelihood of the over- and under-protection of different patients/participants’ rights forewarned by Kass and others. By emphasising overlap, we might also better facilitate responsible innovation and ensure that appropriately-tailored safeguards are in place. In what follows, I suggest the anthropological concept of liminality aids us in this endeavour. More specifically, it does so through the emphasis it places on capturing process, experience and transformation through and across thresholds of human experience. Building upon previous contributions on the value of liminality in health research regulation, I suggest that liminality provides a useful paradigm through which to explore medical innovation, the areas of overlap with practice/treatment and research, and is thereby a first step towards delivering more effective and responsible regulation.

Laurie and others have advanced a framework for a processual approach to health research regulation, grounded in the anthropological concept of ‘liminality’.^79^ The term, derived from the Latin ‘limen’ meaning threshold, was initially developed by anthropologist Arnold van Gennep in *Rites of Passage* in which he draws upon various rituals from diverse cultures in order to explain the human transformative process by which the status of an individual morphs from one status to the other through the crossing of thresholds. The archetypal example is transition from childhood to adulthood and associated rituals related to that social change. Subsequent contributions from Victor Turner in the 1960s built upon liminality, suggesting that it accounts for a state of ‘in betweeness’ where an individual may simultaneously occupy different liminal spaces i.e. both childhood and adulthood, or in fact, they may stop at a space ‘betwixt and between’,^80^ occupying neither status. The exact point at which this transformation may occur may not necessarily be clear. Van Gennep identified three key stages within this process and Turner built upon van Gennep's identification as follows: (1) In the *pre-liminal* phase, the individual is removed from their current status in society – they experience a separation from previous practices and routines; (2) during the *liminal* phase the individual is subject to certain rites or rituals conducted ‘under the authority of a master of ceremonies’. The individual experiences a change in identity; and this implies an actual passing through the threshold that marks the boundary between two phases, the term ‘liminality’ was introduced in order to characterise this passage; and (3) in the *post-liminal* phase the individual emerges with a different status, having undergone a transformation, and is re-incorporated into society.

But what has this got to do with medical innovation? In building upon this concept, Laurie and others (including this author) have outlined the underpinnings of liminality in relation to the value which it might bring to supporting actors navigating some of the ‘liminal spaces’ of health research regulation.^81^ We suggest that liminality and a processual framework ‘challenges us to engage with the processual and experiential dynamics of research, including the ways in which practices, people, and entities are affected by regulation’.^82^ In particular, we emphasise the importance of capturing different experiences of actors and processes involved in these spaces, perspectives which, we argue, have hitherto been overlooked in regulatory approaches to health research, being under or un-accounted for.^83^

Developing on these foundations, this article further contributes to the literature by suggesting that medical innovation represents one such liminal space and that it is typified by multiple thresholds that must be successfully crossed in order for the enterprise to be a success. As such, thinking with liminality helps us to engage better with the processual and experiential dynamics of medical innovation. In the discussion that follows, several examples highlight the value which a liminal lens brings to better understanding medical innovation. It should be noted that the intention is not to consider in detail which regulatory mechanisms might most appropriately regulate such activities. Rather, the value of this analysis lies in the assertion that in order to effectively regulate activities (and select the most appropriate regulatory mechanisms) we need to first understand the spaces which need to be regulated and navigated. In particular, I suggest that liminality helps us to identify an important, yet overlooked, feature of the medical innovation terrain: its twin objectives concerned with *both* individual benefit (typically stressed in the practice/treatment context) *and* wider societal benefit (typically stressed in the research and medical innovation contexts). Thus, a further contribution of this piece lies in highlighting how liminality helps us to understand how we might support key actors in navigating across such complex spaces. An integral component of such an approach lies in resisting law's tendency to automatically fixate upon further prescription and categorisation, often in the form of legislative text. Rather, I suggest that an important first step – provided by liminality – lies in moving *from text to context*. In other words, whilst further regulation, including potential for new legislation or guidance (text), may be necessary, this will only be effective to the extent that it reflects the experiences of actors and the environments within which such activities take place (context), and once it is well understood that existing regulatory spaces are inadequate for the task at hand.

A pertinent aspect of liminality is its concern with transformation. Turner defines this ‘ … as structuring and structured processes and experiences that occur in institutionally delineated time-spaces’.^84^ Turner suggested that the ‘inter-structural’ represents an acknowledgement of ‘the coincidence of opposite processes and notions’ and ‘that which is neither this nor that, and yet is both’.^85^ In other words, two processes can simultaneously exist despite opposition. Within health research, consider the ways in which law constructs the regulation of data and tissue (namely under the General Data Protection Regulation^86^ and the Human Tissue Act 2004). These legal structures do not reflect the realities that tissue is in fact, upon analysis, data. This suggests that in regulation we tend to set up structures that seek an either/or labelling, and thereby fail to reflect ontological coexistence of regulated subject matter in the real world: i.e. research material can be both tissue and data at the same time. There is some evidence in case law of a move towards recognising this reality; *S and Marper v UK*^87^ recognised this relationship between DNA samples and data (and privacy implications) in ways that legislation does not necessarily do so. Indeed, the recently introduced General Data Protection Regulation does not explicitly state that DNA constitutes data for the purposes of the law. However, from a scientific perspective, the value arising from DNA relies precisely on the potential to extract data from it. Thus, these artificial and opposing structures which Turner observed are apparent within health regulation.

Returning to an earlier example, while the traditional locus of the distinction between practice/treatment (benefit of the individual patient) is often set up against research (the pursuit of generalisable knowledge), numerous examples highlight the blurring distinctions between the two: medical innovation is precisely an instance of these activities. Indeed, the field of regenerative medicine and thoracic surgeon Paolo Macchiarini's ‘failed experiments’ provide a stark example of the ambiguities surrounding classification and the potential dangers of forcing these activities into rigid pre-existing regulatory frameworks. It also illustrates the risks of such activities remaining insufficiently accounted for and as McHale puts it, ‘falling through the cracks’.^88^ A Karolinska University Hospital-commissioned external inquiry (the Asplund Report) suggested that the operations which involved transplantation of synthetic tracheas developed by Macchiarini and his team were not categorised as ‘research’ and thus were not subject to procedures for research study ethics approval at Karolinska. Rather, the local ethics committee considered them to constitute medical care ethics^89^ under compassionate use (i.e. medical treatment for humanitarian reasons rather than research).^90^ This was so despite the fact that the synthetic scaffolds used in the procedures were considered to be Advanced Therapy Medicinal Products, and thus subject to approval requirements from the Swedish Medicinal Products Agency.^91^ The Asplund Report concluded that had Macchiarini's operations been correctly classified as clinical research and subject to ethical review, it was ‘very unlikely that the transplants would have been approved’.^92^ It is interesting to note that the initial outcomes of the procedures were reported as *research*, including in *The Lancet*^93^ which has now retracted two papers by Macchiarini and co-authors, with reference to the fact that their research ‘constitutes scientific misconduct’.^94^ Another external inquiry conducted by the Karolinska Institute University Board acknowledged the close connections between the activities carried out at the hospital and the research conducted at the University which led to confusion. Again, the relative uncertainty around classification of these activities illustrates how medical innovation can be variously conceived of as straddling both research and practice/treatment activities. Viewed in this light, an approach inspired by the inter-structural nature of liminality enables us to break down false/forced distinctions and explicitly acknowledge that multiple activities and intentions may coincide. The liminal lens makes more explicit the reality that a given activity can be more than one thing at once; this alerts us to the dangers of regulatory silos. It also suggests we need more overt systems for capturing this and for navigating multiple regulatory spaces at the same time. I will use the database for innovative treatments, as envisioned by s2(1) of AMTIA,^95^ to illustrate this point.

Miola considers in this issue the various challenges associated with the establishment, maintenance and potential utility of any such register. Despite the considerable uncertainties around it (including the fact that the register has still to be established), the question of pursuing a register *through law* provides an interesting example for liminality and its usefulness. Liminality invites us to conceptualise medical innovation as a space which breaks down pre-existing structures and in so doing our approach can better accommodate the co-existence of overlapping activities. As mentioned previously, practice/treatment and research are often differentiated based on whether the primary intention of the activity is to benefit an individual or to generate generalisable knowledge, ultimately leading to wider societal benefits. Each activity triggers distinct regulatory pathways. But as the discussion has demonstrated, this distinction is challenged by the practical realities associated with conducting such activities. A liminal approach that stresses the importance of context and which actively engages with – and at times embraces – anti-structure, allows us to destabilise such distinctions. It helps us acknowledge that medical innovation is a space within which *both* aims of individual benefit and contributing to the wider stock of knowledge can co-exist. In practical terms, such a ‘shared space’ could be conceived of in the form of a register (or other means of robustly capturing the outcomes of innovation). A database could hold the results (positive and negative) of individual responses to innovative interventions and at the same time allow such results to be used to inform other prospective individual treatments as well as further, more robust, study for the purpose of wider knowledge generation for societal benefit. Thus, simultaneously, a register can *protect* individuals (more information available to enrich consent about likely outcomes from previous interventions) and promote new and responsible innovation/research (deliver both a dynamic evidence base and a platform on which to build future protocols).

The previous discussion demonstrates the clear relevance of ‘liminal spaces’ to the medical innovation setting. It has also suggested how a liminal approach might help us to accommodate such coincidence. But, again, with its emphasis on *context,* liminality provides even further valuable insights. An independent inquiry into regenerative medicine research – a field where innovation is particularly prevalent^96^ – noted that contracts between academics and NHS hospitals are commonplace in the UK and can exacerbate the difficulties in determining how to classify processes. The panel explained:

with novel or experimental treatments, the boundaries between what should be classified as academic activity and what should be classified as health service related activity might become blurred. Coupled with this, there can be uncertainty or ambiguity around which organisation's governance framework applies when activities span both (or indeed multiple) institutions.^97^

The report also suggested that added complexity lies in the fact that medicinal products may also be *manufactured*, thus

the boundaries between academic research, manufacturing and clinical treatment may become blurred, [further], where there are different governance structures within different organisations, different nomenclature in use, and different levels of understanding of the quality and regulatory requirements between different partners, then communication and end to end oversight might be compromised.^98^

Yet another report exploring the Karolinska University relationship with Macchiarini found that ‘collaboration confused the division of responsibilities between the university and the hospital, not least the responsibility for ensuring that the required permits were obtained.’.^99^ The changing (and confusing) environment in which such activities take place is of concern elsewhere; in the United States Klein and Fleischman note that ‘the pharmaceutical industry has turned to commercially oriented networks of physicians practicing in private offices’.^100^ Thus, liminality can encourage us to look beyond regulation alone, and to account for the nature of employment of actors involved in ‘innovating’ *across and between different structures* occupying the innovation landscape. Again, it helps us to move from text to context. We must also consider, as Chan has noted, ‘the shifting relationships between different stakeholder groups, the global politics of research and innovation, and the evolving role of publics and patients with respect to science’.^101^

These inter-structural spaces which actors must navigate also imply the potential for transformation across different thresholds; such boundary crossings are experiences which liminality encourages us to account for. For example, the medical professional may ‘transform’ from 'clinician' responsible for direct care whose primary concerns relate to the best interests of the individual patient to 'investigator' who is first and foremost – but not exclusively – preoccupied with knowledge production and scientific rigour outlined within a research protocol; it may not necessarily be clear at which point this transformation takes place. Similar challenges appear in the public health emergency setting where the pressure to innovate may be particularly pressing. Questions arise as to whether first responders are acting in the capacity of humanitarian workers or researchers.^102^ The former role implies that the priority activity is practice/treatment as opposed to the researcher priority of knowledge generation. Relatedly, the individual who seeks medical treatment from her physician may suddenly ‘morph’ into a research participant – potentially unaware or confused about how participation might affect her treatment and the possibility of not receiving the best available treatment (if she receives any treatment at all). When we introduce market considerations, important ethical issues around the patient-participant-consumer also emerge.^103^ Furthermore, given the rise in patient participation, there may be instances where the research team joins the patient-participant as a co-investigator,^104^ thus performing various roles and crossing various regulatory thresholds depending on their activity. Likewise, as considered earlier, the researcher, having worked on the assumption that her obligations are limited to answering a research hypothesis by virtue of incidental findings, may now find herself performing a role more closely in tune with someone responsible for the patients’ clinical care. When we stop to reflect upon the experiences of these actors, it becomes apparent that we must better account for this duality of roles and support actors as they navigate these complex regulatory landscapes. Elsewhere, colleagues have examined the ways in which a liminal approach can assist in recasting the notion of social value in research. Core components of this are precisely focussed on engaging with the experiences of actors across the regulatory research endeavour, and iterative collaboration amongst actors in developing new governance frameworks. The authors stress the importance of incorporating feedback loops which reflect the experiences of actors on the ground (such as researchers) and their subsequent incorporation within regulation.^105^ In the medical innovation context, this could be achieved through conducting more empirical research with the variety of stakeholders involved in doing, experiencing and regulating innovation. In turn, this would involve explicitly folding-in these experiences, within current regulatory responses. One way of doing so, as I have argued elsewhere, is through the inclusion of best-practice alongside pre-existing (often overly abstract) guidance. This provides an important means of supporting decision-makers in interpreting guidance through the use of concrete examples which reflect practical experiences of those actors charged with navigating the regulatory framework.^106^

In the medical innovation setting, some helpful insights from pre-existing empirical work (and which could be reflected within regulatory frameworks) merit consideration. For example, in the context of surgical innovation, it is suggested that there is lack of agreement about which procedures are considered innovative^107^ (in contrast with ‘variations in practice’ and ‘research’). Pace stresses the importance of differentiating between experimental innovation (where a ‘completely new intervention is being attempted’) and personal innovation (where ‘the proposed technique has already been established but the practitioner concerned has either never attempted it before or is still learning how to perform it’).^108^ Interviews conducted by Rogers and others with surgeons reveal that first time attempts of a pre-existing technique may or may not constitute innovation to those performing them.^109^ Rogers and others also found that surgeons lacked consensus around the relationship between research and innovation with some interviewees suggesting that innovation is a type of research, some suggesting they are quite separate activities, some describing research as preceding innovation and others supporting the reverse.^110^ Further, there may be an unwillingness amongst surgeons to categorise their activities as ‘innovative’ due to the associated regulatory oversight, with tendency to label activities as ‘research’ for the purposes of publication.^111^ It is important to consider whether difficulties in delineation arise due to a gap between stated intention (to benefit a patient) and true intention (advancing research whilst avoiding a potentially tougher regulatory framework).^112^ Likewise, particularly in the context of academic clinicians/researchers, the pressures of a publish or perish culture must also be considered. Additionally, Hutchison and others stress that leaving it up to surgeons to determine whether or not their activities constitute ‘innovation’ has substantial implications for patient safety: ‘patients are exposed to risks, with sometimes tragic consequences’.^113^

One final aspect of liminality might assist us in avoiding the risks associated with leaving categorisation of activities to the discretion of one individual, team or institution. Liminality suggests that transformation often takes place under the authority of a ‘Master of Ceremonies’ (MoCs) or ‘Representative of Order’ (RoO). In human societies it is common that actors (for example, priests, shamen, village elders) are charged with overseeing rituals (e.g. christenings, marriages, tribe initiations) and in guiding the individual across thresholds, as they undergo transformation.^114^ In the health research regulation context, I have argued with colleagues elsewhere that there are parallels to be found in the notion of regulatory stewardship.^115^ Examples of regulatory stewardship in action include NHS Research Ethics Committees and HRA Application Managers.^116^ These bodies guide researchers through the regulatory landscape. Turning to the context of medical innovation, a role for regulatory stewards might lie in the creation of individual roles or teams tasked with supporting individuals in determining whether activities ought to be categorised under practice/treatment, research and/or innovation. Such actors could work alongside regulators and outwith the institutions within which the activities are taking place. Regulatory stewards might also be involved in the establishment and maintenance of a register of medical innovations, advising on data capture and facilitating sharing arrangements. These are only preliminary illustrations, as more work is needed in both developing the notion of regulatory stewardship and exploring the legal avenue of creating a register of medical innovation. But, true to the lessons from liminality, the law alone cannot provide such benefits, humans must be led through processes of transition and change.^117^ This analysis offers us helpful starting points in developing a more holistic conceptualisation of medical innovation and in understanding how we can better support actors as they attempt to navigate their way across these sites of transformation and blurred boundaries.

## 5. Conclusion

This article has highlighted the pressing need to develop a more holistic conceptualisation of medical innovation in order to be better able to determine which regulatory approaches might be appropriate to govern such activities. It has asked if, and how, the anthropological concept of liminality might serve to enrich our understanding of medical innovation and by consequence, the relationships between innovation, practice/treatment and research. Some challenges in developing a satisfactory definition of medical innovation centre around the need to simultaneously distinguish it from research and practice/treatment whilst also to accommodate areas of overlap between all three – this challenge has not yet received the attention it deserves. Further, a liminal approach allows us to engage with the inter-structural dynamics at play as an alternative to law's tendency to categorise and silo activities, creating false and unhelpful distinctions. Liminality also invites us to account for the experiences of processes, actors and things involved in medical innovation; the examples provided demonstrate that many such aspects that shape medical innovation are under or un-accounted for within pre-existing conceptualisations, presenting significant challenges for regulation. Important synergies also emerge between liminality's preoccupation with transformation, and the various changes of status that are evident across the medical innovation landscape. By these means, the discussion has highlighted the pressing need for further work, both empirical and conceptual, in order to better understand the activities we seek to regulate.

## Footnotes

1 Bernard Dickens, ‘Human Rights in Medical Experimentation’ (1979) 9(23) *Israel Yearbook on Human Rights* 23.

2 UK Department of Health, ‘Accelerated Access Review: Final Report Review of innovative medicines and medical technologies’ (October 2016) www.gov.uk/government/uploads/system/uploads/attachment_data/file/565072/AAR_final.pdf p10 (this and all other links in this article were last accessed on 28 August 2018).

3 The Lancet Neurology (Editorial), ‘Increasing the Power of Public Awareness Campaigns’ (2015) 14(7) *The Lancet Neurology* 669.

4 Jonathan Darrow and others, ‘Practical, Legal, and Ethical Issues in Expanded Access to Investigational Drugs’ (2015) 372 *New England Journal of Medicine* 279.

5 Tim Mackey and Virginia Schoenfeld, ‘Going “Social” to Access Experimental and Potentially Life-Saving Treatment: An Assessment of the Policy and Online Patient Advocacy Environment for Expanded Access’ (2016) 14 *BMC Medicine* 17; Arthur Caplan and Kenneth Moch, ‘Rescue Me: The Challenge of Compassionate Use in the Social Media Era’ (2014) Bethesda Health Affairs Blog http://healthaffairs.org/blog/2014/08/27/rescue-me-the-challenge-of-compassionate-use-in-the-social-media-era/.

6 Denise Meyerson, ‘Medical Negligence Determinations, the “Right to Try,” and Expanded Access to Innovative Treatments’ (2017) 14(3) *Journal of Bioethical Inquiry* 385; David Vulcano, ‘The Regulatory Debate around Pre-approval Product Access’ (2017) 26(4) *Applied Clinical Trials* 30.

7 José Miola, ‘Bye-Bye Bolitho? The Curious Case of the Medical Innovation Bill’ (2015) 15 (2–3) *Medical Law International* 124; José Miola and Nils Hoppe, ‘Innovation in Medicine Through Degeneration in Law? A Critical Perspective on the Medical Innovation Bill’ (2014) 14(4) *Medical Law International* 266; Bernadette Richards, ‘Medical Innovation Laws: An Unnecessary Innovation’ (2015) 40(3) *Australian Health Review* 282.

8 World Health Organization, ‘Progress Report on the Development of the WHO Health Emergencies Programme’ (2016) www.who.int/about/who_reform/emergency-capacities/who-health-emergencies-programmeprogress-report-march-2016.pdf.

9 Gov.UK, ‘SBRI Funding Competition: Vaccines for Global Epidemics’ (8 November 2016) www.gov.uk/government/publications/sbri-funding-competition-vaccines-for-global-epidemics-clinical.

10 Etienne Vignola-Gagné, Peter Biegelbauer and Daniel Lehner, ‘Translational Research: Entrepreneurship, Advocacy and Programmatic Work in the Governance of Biomedical Innovation’ in S. Borrás and J. Edler (eds), *The Governance of Socio-Technical Systems. Explaining Change* (Edward Elgar Publishing 2014) 132; John Gardner and Andrew Webster, ‘The Social Management of Biomedical Novelty: Facilitating Translation in Regenerative Medicine’ (2016) 156 *Social Science & Medicine* 90; NHS England www.england.nhs.uk/ourwork/innovation/test-beds/.

11 See Accelerated Access Collaborative: www.nice.org.uk/aac.

12 See Innovative Medicines Initiative: www.imi.europa.eu/; European Institute of Innovation and Technology, Innovation Communities: https://eit.europa.eu/activities/innovation-communities.

13 European Commission, Responsible Research and Innovation (2012): http://ec.europa.eu/research/science-society/document_library/pdf_06/responsible-research-and-innovation-leaflet_en.pdf.

14 Graeme Laurie, ‘Liminality and the Limits of the Law in Health Research Regulation’ (2016) 25(1) *Medical Law Review* 47; Samuel Taylor-Alexander and others, ‘Beyond Regulatory Compression: Confronting the Liminal Spaces of Health Research Regulation’ (2016) 8(2) *Law, Innovation and Technology* 149, 175.

15 Nayha Sethi and others, Liminal Spaces Workshop: Regulating for Uncertainty. 1-2 February 2018, Wellcome, London.

16 Institute of Medicine, Roundtable on Evidence-Based Medicine, LeighAnne Olsen, Dara Aisner and Michael McGinnis (eds), *The Learning Healthcare System: Workshop Summary* (National Academies Press 2007) 6.

17 Nancy Kass and others, ‘The Research-Treatment Distinction: A Problematic Approach for Determining Which Activities Should Have Ethical Oversight’ (2013) 43(1) *Hastings Center Report Special Report* 4. https://doi.org/10.1002/hast.134.

18 Bosse Linquevist documentary ‘The Experiments’ www.svt.se/dokument-inifran/experimenten-in-english/ aired in the UK by the BBC as ‘Fatal Experiments: Downfall of a Surgeon’: www.bbc.co.uk/programmes/b080k2z4.

19 Kjell Asplund, ‘Macchiarini Fallet Investigation of the Activities of Transplantation of Synthetic Trachea Karolinska University Hospital Report’ (31 August 2016) [14]. www.circare.org/info/pm/fallet-macch-google-20160903.pdf.

20 Medical practice may refer to all that it entails to be a medical professional whereas treatment may be construed to refer to the specific administering of treatment to an individual.

21 Based in part on the distinctions offered by Robert Levine, ‘The Boundaries Between Biomedical or Behavioral Research and the Accepted and Routine Practice of Medicine’ The Belmont Report, Appendix (1978) Volume 1, DHEW Pub. No. (OS) 78-0013, U.S. Printing Office).

22 National Commission for the Protection of Human Subjects of Biomedical and Behavioral Research, *Ethical Principles and Guidelines for the Protection of Human Subjects of Research* (1978).

23 Ibid.

24 Ibid at Part A.

25 For example, World Medical Association Declaration of Helsinki (2013); Council for International Organizations of Medical Research, International Ethical Guidelines for Health-related Research Involving Humans (2016); *Walker-Smith v GMC* [2012] EWHC 503 (Admin). Whilst the court acknowledged the grey area between practice and research, part of the test it put forward for determining whether the professional was guilty of misconduct centred on whether his intention was to benefit the patient or to ‘add to the stock of knowledge’.

26 A comprehensive overview is provided by Jean McHale in this special issue.

27 Bolam v Friern Hospital Management Committee [1957] 1 WLR 582.

28 Academy of Medical Royal Colleges: www.aomrc.org.uk/

29 NHS, ‘Can I Demand a Specific Treatment?’ www.nhs.uk/common-health-questions/nhs-services-and-treatments/can-i-demand-a-specific-treatment/.

30 GMC Ethical Guidance: www.gmc-uk.org/ethical-guidance.

31 Colin Thomson, ‘Research Ethics Committees’ in *Encyclopedia of Applied Ethics Second Edition* (Elsevier 2012).

32 HRA Approvals: www.hra.nhs.uk/approvals-amendments/what-approvals-do-i-need/hra-approval/.

33 MHRA: www.gov.uk/government/organisations/medicines-and-healthcare-products-regulatory-agency.

34 For example the EU Clinical Trials Directive 2001/20/EC.

35 See for example *Walker-Smith* (n 25).

36 Olsen (n 16).

37 Kass and others (n 17) s5.

38 Ibid.

39 Nuffield Council on Bioethics, ‘Conducting Research and Innovation During Global Health Emergencies: What are the Ethical Challenges?’ Notes of workshop held on 9 December 2016: London http://nuffieldbioethics.org/wp-content/uploads/Research-in-GHEs-note-for-web.pdf.

40 Philippe Calain and others, ‘Research Ethics and International Epidemic Response: The Case of Ebola and Marburg Haemorrhagic Fevers’ (2009) 2(1) *Public Health Ethics* 7.

41 For related discussion see Gerard Keusch and others (eds), *Integrating Clinical Research into Epidemic Response: The Ebola Experience.* Report from the National Academy of Sciences (National Academic Press 2017).

42 See for example Susan Wolf and others, ‘Managing Incidental Findings in Human Subjects Research Analysis and Recommendations’ (2008) 36(2) *The Journal of Law, Medicine & Ethics* 219.

43 Benjamin Berkman, Sara Chandros Hull and Lisa Eckstein, ‘The Unintended Implications of Blurring the Line Between Research and Clinical Care in a Genomic Age’ (2014) 11(3) *Personalized Medicine* 28.

44 See for example: John Kenyon Mason and Graeme Laurie, *Mason and McCall Smith's Law and Medical Ethics* (Oxford University Press, 10th edn 2016); Phoebe Friesen and others, ‘Rethinking the Belmont Report?’ (2017) 17(7) *American Journal of Bioethics* 15; Shimon Tashiro, ‘Research, Practice, and Innovative Therapy: On the Theoretical Model of Robert J. Levine’ (2010) 2(3) *Asian Bioethics Review* 229.

45 See: Anahita Baregheh, Jennifer Rowley and Sally Sambrook, ‘Towards a Multidisciplinary Definition of Innovation’ (2009) 47(8) *Management Decision* 1323.

46 Hilary Sutcliffe, ‘A Report on Responsible Research and Innovation’ (2011) https://ec.europa.eu/research/science-society/document_library/pdf_06/rri-report-hilary-sutcliffe_en.pdf.

47 Ibid.

48 Herbert Hart, *The Concept of Law*, Penelope Bulloch and Joseph Raz (eds), (Clarendon Press, 2nd edn 1994).

49 Jeffrey Aronson ‘Something New Every Day Defining Innovation and Innovativeness in Drug Therapy’ (2008) 31(1) *The Journal of Ambulatory Care Management* 65; Kathleen Garnett, Geert Van Calster and Leonie Reins ‘Towards an Innovation Principle: An Industry Trump or Shortening the Odds on Environment’ (2018) *Law, Innovation and Technology* online first DOI: 10.1080/17579961.2018.1455023; Anna Butenko and Pierre Larouche ‘Regulating for Innovativeness or Regulation of Innovation?’ (2015) 7(1) *Law, Innovation and Technology* 52.

50 Butenko and Larouche ibid.

51 Aronson (n 49); Robert Edgell and Roland Vogl, ‘A Theory of Innovation: Benefit, Harm, and Legal Regimes’ (2015) 5(1) *Law, Innovation and Technology* 53.

52 Davide Nicolini, ‘Medical Innovation as a Process of Translation: A Case from the Field of Telemedicine’ (2010) 21 *British Journal of Management* 1011.

53 Shawn Harmon, ‘Responsible Regulation in Action? Responsible Research and Innovation and the European Bank for Induced Pluripotent Stem Cells’ (2018) *Law, Innovation and Technology* online first DOI: 10.1080/17579961.2018.1452175; *Simms v Simms* [2003] 2 WLR 1465.

54 Definitional ambiguity associated with the term will be discussed further below.

55 For discussion see Jean McHale's contribution in this special issue.

56 Also referred to as ‘expanded access’ schemes.

57 Gayathri Balasubramanian and others, ‘An Overview of Compassionate Use Programs in the European Union Member States’ (2016) 5(4) *Intractable & Rare Diseases Research* 244.

58 MHRA: www.gov.uk/guidance/advanced-therapy-medicinal-products-regulation-and-licensing.

59 Tsachi Keren-Paz and Alicia El Haj, ‘Liability Versus Innovation: The Legal Case for Regenerative Medicine’ (2014) *Tissue Engineering Part A*, 1 DOI: 10.1089/ten.tea.2013.0324; see also Tsachi Keren-Paz's contribution in this special issue.

60 The latter includes the desirability of follow up by a research programme where the innovation was not initially performed as research.

61 Johanna Hanefeld, Richard Smith, and Thinakorn Noree, ‘Medical Tourism’ (2016) *World Scientific Handbook of Global Health Economics and Public Policy* 333.

62 European Commission, Drivers of International Collaboration in Research (2009) EUR 24195 EN.

63 Jan Borysowski, Hans-Jörg Ehni and Andrzej Górski, ‘Ethics Review in Compassionate Use’ (2017) 15 *BMC Medicine* 136.

64 Tamra Lysaght and others, ‘Regulatory Challenges with Autologous Adult Stem Cells: A Comparative Review of International Regulations’ (2017) 14 *Bioethical Inquiry* 261.

65 House of Commons Science and Technology Committee Report, *Regenerative Medicine* 2016–2017 HC 275.

66 For an extensive overview of the varying terminology in this area see Nicholas Pace, ‘The Regulation of Innovation’, (PhD Thesis, Institute of Law and Financial Studies University of Glasgow 2005).

67 Wendy Rogers and others, ‘Identifying Surgical Innovation: A Qualitative Study of Surgeons’ Views’ (2014) 259 *Annals of Surgery* 273.

68 Lysaght and others (n 58).

69 Aronson (n 49).

70 Ch 9.

71 Tracey Chan, ‘Legal and Regulatory Responses to Innovative Treatment’, (2013) 21(1) *Medical Law Review* 92. Emphasis added.

72 Dale Cowan, ‘Innovative Therapy Versus Experimentation’ (1985) 169 *Torts Ins L J* 619, 623.

73 Often but not exclusively in the form of clinical trials.

74 Various versions of the translational continuum have been discussed. See for example Giulio Cossu and others, ‘The Lancet Commission: Stem Cells and Regenerative Medicine’ (2018) 391(10123) *The Lancet* 883; Brian Drolet and Nancy Lorenzi, ‘Translational Research: Understanding the Continuum from Bench to Bedside’ (2011) 157(1) *Translational Research* 1.

75 Keren-Paz and El Haj (n 59).

76 Lyria Bennett Moses, ‘Recurring Dilemmas: The Law's Race to Keep Up with Technological Change’ (2007) 7 *University of Illinois Journal of Law, Technology and Policy* 239.

77 Taylor-Alexander and others (n 14) 175.

78 Nayha Sethi, ‘Reimagining Regulatory Approaches: On the Essential Role of Principles in Health Research Regulation’ (2015) 12 *SCRIPTed* 91.

79 Laurie (n 14); Taylor-Alexander and others (n 14); Agomoni Ganguli Mitra and others, ‘Reconfiguring Social Value in Health Research Through the Lens of Liminality’ (2017) 31(2) *Bioethics*https://doi.org/10.1111/bioe.12324.

80 Victor Turner, *Ritual Process: Structure and Anti-Structure* (Cornell University Press, 1969) 95.

81 Laurie (n 14); Taylor-Alexander and others (n 14); Ganguli-Mitra and others (n 79).

82 Taylor-Alexander and others (n 14).

83 Laurie (n 14).

84 (n 80).

85 Ibid 7.

86 Regulation (EU) 2016/679 of the European Parliament and of the Council of 27 April 2016 on the protection of natural persons with regard to the processing of personal data and on the free movement of such data, and repealing Directive 95/46/EC (General Data Protection Regulation).

87 (2009) 48 EHRR.

88 See Jean McHale's contribution in this special issue.

89 Maureen McKelvey, Rögnvaldur Saemundsson and Olof Zaring, ‘A Recent Crisis in Regenerative Medicine: Analyzing Governance in Order to Identify Public Policy Issues’ (2018) *Science and Public Policy* scx085, https://doi.org/10.1093/scipol/scx085.

90 Asplund (n 19).

91 These approvals were not sought. See McKelvey and others (n 89).

92 Asplund (n 19) [14].

93 Phillip Jungebluth and others, ‘Tracheobronchial Transplantation with a Stem-Cell-Seeded Bioartificial Nanocomposite: A Proof-of-Concept Study’ (2011) 378 *The Lancet* 1997.

94 Editorial, ‘The Final Verdict on Paolo Macchiarini: Guilty of Misconduct’ (2018) 392(10141) *The Lancet* 2.

95 (n 70). The Act, and its various controversial earlier incarnations including the Medical Innovation Bill (Saatch Bill), are considered by José Miola's contribution in this special issue, who takes a more critical approach of the database than offered above.

96 Giulio Cossu and others, ‘The Lancet Commission: Stem Cells and Regenerative Medicine’ (2018) 391(10123) *The Lancet* 883.

97 Stephen Wigmore and others, Special Inquiry into Regenerative Medicine Research at UCL (September 2017) 9. www.ucl.ac.uk/news/news-articles/0917/Special_Inquiry_Final_Report_605109702_7_.pdf.

98 Ibid 16.

99 Sten Heckscher, Ingrid Carlberg and Carl Gahmberg, ‘Karolinksa Institutet and the Macchiarini Case. Summary in Swedish and English’, (2016) https://ki.se/sites/default/files/karolinska_institutet_and_the_macchiarini_case_summary_in_english_and_swedish.pdf.

100 Jason Klein and Alan Fleischman, ‘The Private Practicing Physician-Investigator: Ethical Implications of Clinical Research in the Office Setting’ (2002) 32 *Hastings Center Report* 22.

101 Sarah Chan, ‘Current and Emerging Global Themes in the Bioethics of Regenerative Medicine: The Tangled Web of Stem Cell Translation’ (2017) 12(7) *Regenerative Medicine* 839.

102 Agomoni Ganguli Mitra and Nayha Sethi, ‘Conducting Research in the Context of Global Health Emergencies: Identifying Key Ethical and Governance Issues’ (Nuffield Council on Bioethics, 2016).

103 Chan (n 101).

104 See Michelle Robbins, Janice Tufte and Clarissa Hsu, ‘Learning to “Swim” with the Experts: Experiences of Two Patient Co-investigators for a Project Funded by the Patient-Centered Outcomes Research Institute’ (2016) 20(2) *The Permanente Journal* 88.

105 Ganguli Mitra and others (n 79).

106 Nayha Sethi, ‘Research and Global Health Emergencies: On the Essential Role of Best Practice (2018) *Public Health Ethics* phy014 https://doi.org/10.1093/phe/phy014

107 Rogers and others (n 67).

108 Pace (n 60).

109 Rogers and others (n 67).

110 Ibid.

111 Angelique Reitsma and Jonathan Moreno, ‘Ethics of Innovative Surgery: U.S. Surgeons’ Definitions, Knowledge and Attitudes’ (2005) 200(1) *Journal of the American College of Surgeons* 103.

112 See Tsachi Keren-Paz's contribution in this special issue.

113 Katrina Hutchison and others, ‘Getting Clearer about Surgical Innovation: A New Definition and a New Tool to Support Responsible Practice’ (2015) 262(6) *Annals of Surgery* 949.

114 Arpad Szakolczai, ‘Liminality and Experience: Structuring Transitory Situations and Transformative Events’ in Agnes Hovarth, Bjørn Thomassen and Harald Wydra (eds), *Breaking Boundaries: Varieties of Liminality* (Berghahn Books 2015) 18.

115 Graeme Laurie and others, ‘Charting Regulatory Stewardship in Health Research: Making the Invisible Visible’, (2018) 27 (2) *Cambridge Healthcare Quarterly* 333.

116 Ibid.

117 As the work on liminality has shown, a dynamic continuum – informed by liminality and in particular its preoccupation with thresholds – offers a helpful conceptual device through which to explore these transitions. Space limits prevent me from elaborating on dynamic continuum here.

